# Effects of Antiseizure Medications on Second-Trimester Prenatal Screening Test Parameters: A Retrospective Cohort Study

**DOI:** 10.3390/medicina61061101

**Published:** 2025-06-17

**Authors:** Melisa Golgelioglu, Cigdem Akcabay, Gunes Seda Albayrak, Selda Telo

**Affiliations:** 1 Department of Obstetrics and Gynaecology, Yozgat City Hospital, 66100 Yozgat, Turkey; 2 Department of Obstetrics and Gynaecology, Faculty of Medicine, Fırat University, 23119 Elazig, Turkey; cagcabay@hotmail.com; 3 Department of Neurology, Faculty of Medicine, Yozgat Bozok University, 66100 Yozgat, Turkey; drgunesalbayrak@yahoo.com; 4 Biochemistry and Clinical Biochemistry, Faculty of Medicine, Fırat University, 23119 Elazig, Turkey; drseldatelo@hotmail.com

**Keywords:** alpha-fetoprotein, fetal anomalies, human chorionic gonadotropin, teratogenicity, unconjugated estriol

## Abstract

*Background and Objectives*: The use of antiseizure medications (ASMs) during pregnancy is critical to seizure control in women with epilepsy but raises concerns regarding the use of these drugs and their possible effect on the maternal serum biochemical markers used for second-trimester prenatal screening. The aim of this study was to assess the effect of ASMs on the levels of maternal serum alpha-fetoprotein (AFP), unconjugated estriol (uE3), and human chorionic gonadotropin (hCG) assessed in the serum biomarker analyses part of second-trimester prenatal screening. *Materials and Methods:* This retrospective cohort study included 43 pregnant women in the ASM-exposed group (levetiracetam, lamotrigine, carbamazepine, or combined therapy) and 43 matched controls without medication use. Groups were matched based on maternal age, gravidity, parity, abortion history, gestational age at testing, body mass index, and smoking status with propensity score matching. Serum AFP, uE3, and hCG levels measured at 15–20 weeks of gestation were compared between groups. The incidence of fetal congenital anomalies or aneuploidies was also compared between groups. *Results*: Pregnant women in the ASM-exposed group had significantly higher maternal serum AFP (1.34 ± 0.42 vs. 1.01 ± 0.31 MoM; *p* < 0.001) and uE3 (1.28 ± 0.39 vs. 1.05 ± 0.34 MoM; *p* = 0.004) than the controls. However, hCG did not differ significantly between the groups (1.07 ± 0.46 vs. 1.01 ± 0.42 MoM; *p* = 0.523). Regarding the ASM subgroups (levetiracetam, lamotrigine, and carbamazepine), there were no significant differences in the serum biomarkers (*p* > 0.05). There was no significant difference between the ASM-exposed and control groups in terms of the incidence of congenital anomalies or aneuploidies (2.3% in the ASM-exposed group vs. 2.3% in the control group; *p* = 1.000). *Conclusions*: The use of ASMs during pregnancy significantly alters second-trimester maternal serum biochemical markers, including our primary concerns, AFP and uE3, which could cause inaccurate interpretations of second-trimester prenatal screening. Clinicians should carefully consider maternal medication exposure when interpreting these biochemical markers in pregnant women with epilepsy to prevent the misclassification of fetal risks and avoid unnecessary invasive procedures.

## 1. Introduction

Epilepsy is one of the most prevalent neurological diseases during pregnancy, affecting approximately 0.3–0.7% of pregnant women worldwide [1]. Management typically involves the use of antiseizure medications (ASMs), essential to seizure control; however, their use raises concerns due to potential teratogenic effects [2]. Uncontrolled seizures during pregnancy have been associated with adverse obstetric outcomes, such as miscarriage, stillbirth, preterm delivery, and developmental delays in offspring, emphasizing the critical need for effective seizure control [3,4]. Previous retrospective studies have identified significant alterations in maternal serum biomarkers used in prenatal screening tests among women treated with ASMs, highlighting an important clinical consideration when interpreting these biomarkers [5,6]. Given the complex interplay among maternal ASM exposure, fetal risk, and biomarker levels, precise prenatal screening and the accurate interpretation of test results are essential. There remains a critical area of clinical research regarding the influence of ASM therapy on maternal–fetal health outcomes.

Maternal serum screening tests to screen for aneuploidies and neural tube defects in fetuses during the second trimester are now performed routinely. The tests routinely examine markers of fetal origin, with the aim of quantifying maternal serum concentrations of biomarkers including alpha-fetoprotein (AFP), unconjugated estriol (uE3), and human chorionic gonadotropin (hCG) [7]. Previous studies investigating the effects of ASMs on prenatal serum screening parameters have yielded varying results. For instance, Öksüzoğlu et al. [5] reported significant elevations in AFP and uE3 levels among women using ASMs compared with controls, and Besimoğlu et al. [6] similarly observed increased biochemical markers (AFP and uE3) without changes in hCG. The importance of these biomarker levels in maternal serum as potential indicators of fetal anomalies or genetic conditions and for assessing potential adverse maternal–fetal outcomes in pregnancy cannot be underestimated. This means that the careful interpretation of these tests is important in clinical practice because they can inform important decisions regarding further diagnostic investigations with invasive diagnostic procedures and care options in pregnancy [8,9,10]. The misinterpretation of these biomarkers can lead to unnecessary procedures, increased maternal anxiety and distress, and the potential cost of additional health care [8,9,10]. Therefore, it is vital that clinical factors that may influence marker levels in serum are carefully scrutinized to ensure sound clinical decision-making.

The hypothesis underpinning this study is predicated upon increasing although limited evidence suggesting that ASMs can impact maternal serum biochemical marker concentrations via multiple mechanisms of action. First, ASMs can induce hepatic microsomal enzymes, specifically, the cytochrome P450 enzyme system, which is involved in the metabolism and clearance of hormones. Previous studies have shown that carbamazepine, valproic acid, and levetiracetam give rise to asignificant activation of hepatic enzymes [11,12], which may cause maternal serum concentrations of hormones such as AFP, uE3, and hCG to be altered. Second, the direct effects of valproic acid and levetiracetam on hormone secretion by trophoblastic cells have been documented, which is further evidence of their potential impact on biochemical markers utilized for prenatal screening [13]. Despite these indications, clinical studies investigating the effects of ASMs on second-trimester maternal serum screening tests are limited in number, are often methodologically heterogeneous, and involve relatively small sample sizes [6,7].

Thus, the current study’s main focus is to determine if the use of ASMs has an impact on maternal serum biochemical marker levels in screening tests during the second trimester. In this study, we aim to clarify these potential impacts to improve the clinical interpretation of screening test results, reduce unnecessary invasive procedures, and optimize prenatal management in pregnant women with epilepsy.

## 2. Materials and Methods

### 2.1. Study Design

This study was designed as a retrospective cohort analysis conducted at the obstetrics and gynecology departments of three hospitals (two tertiary care centers and one secondary care center). Data related to ASM use, pregnancy follow-up, antenatal visits, medication adherence, seizure frequency, obstetric assessments, pregnancy outcomes, and second-trimester maternal serum screening test results were retrieved from existing hospital medical records and patient files. This study commenced after receiving approval from the Fırat University Institutional Review Board (approval date: 1 November 2024; approval number: 28573). All procedures performed in this study adhered to the ethical standards of the institutional and national research committee, as well as the principles outlined in the Declaration of Helsinki for medical research involving human subjects.

This retrospective cohort design was selected due to its effectiveness in evaluating potential medication effects using existing clinical data, thereby minimizing patient-related risks and ethical concerns. The second trimester was specifically chosen because maternal serum biochemical markers used for prenatal screening (AFP, uE3, and hCG) are routinely measured and clinically interpreted during this period to screen for fetal anomalies, particularly neural tube defects and aneuploidies, and these screening protocols have been extensively validated and are routinely utilized worldwide [7]. Previous similar studies have also demonstrated the validity and utility of analyzing second-trimester screening markers in evaluating the impact of ASMs [5,6].

### 2.2. Patient Selection

**Figure 1:**
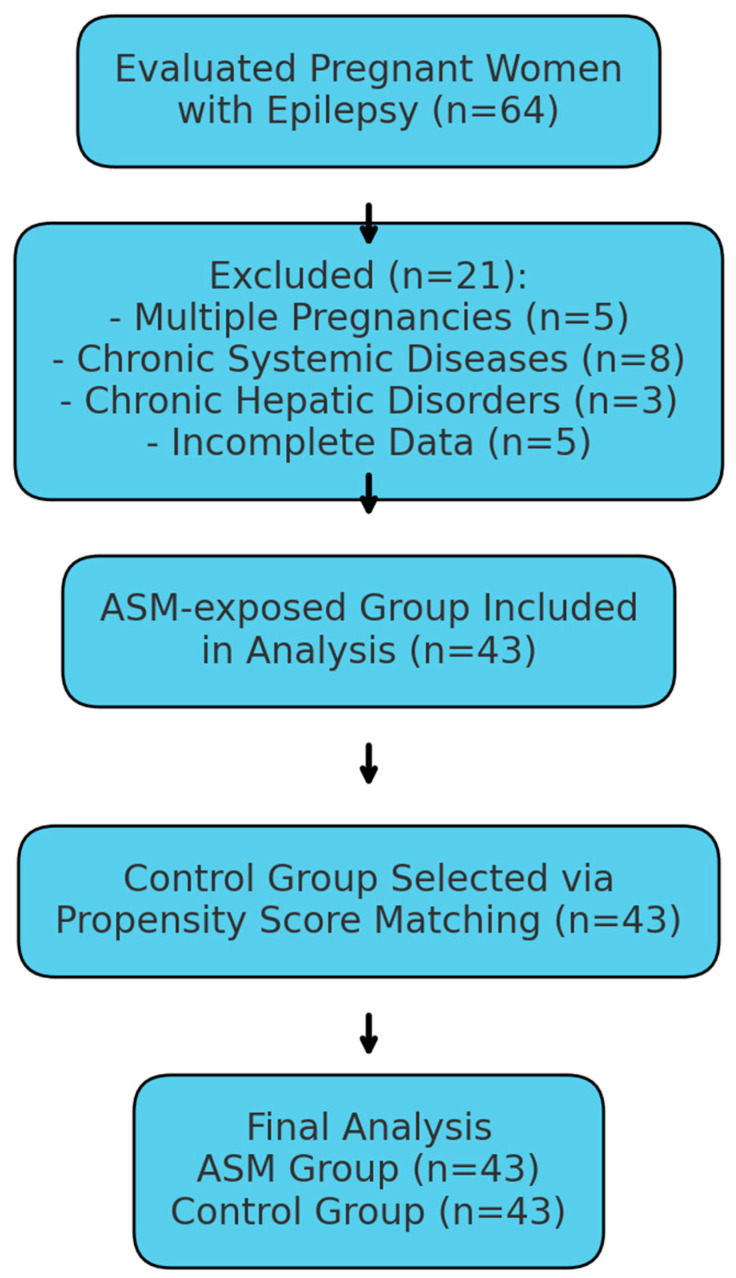
Flow diagram illustrating the participant selection process, exclusion criteria, and final numbers of pregnant women included in the ASM-exposed and control groups.

Medical records of pregnant women with epilepsy who attended our obstetrics and gynecology departments between January 2020 and September 2024 were retrospectively reviewed. Pregnant women aged between 18 and 44 years using ASMs throughout pregnancy and having second-trimester maternal serum screening test results available were included. The exclusion criteria for the epilepsy group were multiple pregnancies, chronic systemic diseases (such as diabetes mellitus, hypertension, or autoimmune disorders), known chronic hepatic disorders (e.g., chronic hepatitis, cirrhosis, and primary biliary cholangitis), and pregnancies with incomplete medical records or unavailable serum screening results. Isolated elevations in the levels of liver enzymes (alanine aminotransferase (ALT) or aspartate aminotransferase (AST)) were not considered exclusion criteria in the epilepsy group, as these elevations could potentially relate to ASM use. Initially, the medical records of 64 pregnant women with epilepsy were evaluated. Among these, 5 patients were excluded due to multiple pregnancies, 8 patients due to chronic systemic diseases (4 with diabetes mellitus, 3 with hypertension, and 1 with autoimmune disorder), 3 patients due to known chronic hepatic disorders, and 5 patients due to incomplete medical records or unavailable serum screening test results. After applying these inclusion and exclusion criteria, a total of 43 patients remained eligible and were included in the analysis. For patients using ASMs, the medication consistently used during at least the three months prior to the second-trimester maternal serum screening test was taken as the basis for the analysis. Figure 1 illustrates the patient selection process, detailing the reasons for exclusion and clearly showing the final number of participants included in the ASM-exposed and control groups.

The control group consisted of pregnant women who did not use any ASMs or other medications. The same inclusion and exclusion criteria applied to control group; however, in the control group, women with elevated liver enzyme levels (ALT or AST greater than two times the upper limit of normal) were also excluded to eliminate potential subclinical hepatic dysfunction. The control patients were matched to the patients in the epilepsy group using propensity score matching with respect to age, gravidity, parity, abortion history, gestational age at testing, BMI, and smoking status [14]. If a patient in the epilepsy group had more than one control candidate available for matching, the candidate with the most similar propensity score to the reference patient was chosen to ensure matched pairs had the least differences [14]. Following propensity score matching, a total of 43 patients were included in each group. These inclusion and exclusion criteria were chosen based on previous studies suggesting these conditions and medications might alter maternal serum biochemical marker levels, potentially confounding the association between ASM use and prenatal screening markers [5,6,7,8,9,10,11].

The diagnosis of epilepsy was established by neurologists according to the clinical history of recurrent seizures, supported by electroencephalography (EEG) and neuroimaging findings. All patients included in this study had previously undergone at least one EEG evaluation confirming epileptiform discharges consistent with epilepsy. Neuroimaging studies, primarily magnetic resonance imaging, were performed at diagnosis or selectively during follow-up in patients with atypical clinical presentations, focal neurological deficits, or suspected structural abnormalities. Common imaging findings included mesial temporal sclerosis and cortical dysplasia or were unremarkable. Specific EEG and neuroimaging results were documented from the patients’ medical records.

### 2.3. Data Collection

Data were collected retrospectively from the electronic medical records stored in the hospital information system and the patient files at the obstetrics and gynecology departments of the participating hospitals. The demographic data of maternal age, gravidity, parity, abortion history, smoking status, and BMI were recorded. Clinical data, including gestational age at the time of second-trimester serum screening (between 15 and 20 weeks of pregnancy), ASM type and dosage, duration of drug use, and maternal systemic diseases, were also obtained from the records. Additionally, second-trimester maternal serum screening test results—including maternal serum AFP, uE3, and hCG—were extracted from the laboratory database. All collected data were independently verified by two investigators, and discrepancies were resolved by consensus, thus ensuring data accuracy and completeness.

### 2.4. Patient Follow-Up

Data related to pregnancy follow-up, including antenatal visits, obstetric assessments, medication adherence, seizure frequency, EEG monitoring, obstetric complications, and pregnancy outcomes, were retrospectively retrieved from hospital medical records and patient files. Clinical follow-up in these patients was conducted according to standard protocols during their pregnancies, and all available data were extracted from these routine clinical records for study purposes. Maternal serum screening tests were performed once during the second trimester (between 15 and 20 weeks of gestation), consistently with standard clinical guidelines.

The patients included in the ASM-exposed group used antiseizure medications consistently throughout pregnancy, starting from at least three months before conception and continuing until delivery. The patients’ seizure control status was routinely assessed at each antenatal visit by a neurologist, documenting seizure frequency, type, and treatment adherence. EEG monitoring was performed selectively when clinically indicated, particularly in cases of suspected changes in seizure frequency or pattern. Epileptic seizures were classified according to the latest 2025 International League Against Epilepsy (ILAE) seizure classification [15]. Additional clinical seizure information recorded included seizure etiology (genetic, structural, infectious, metabolic, immune, or unknown) and semiology (focal, generalized, combined generalized–focal, or unknown onset). Medication dosages were reviewed regularly, and adjustments were made by the neurologist based on seizure control status and side effects. Information on status epilepticus episodes, hospitalizations, emergency room visits, diagnoses of drug-resistant epilepsy, psychiatric comorbidities, and substance abuse was also documented. Routine detailed anatomical ultrasound scans were performed between 18 and 22 weeks of gestation for all participants. Neonatal outcomes, including the presence or absence of congenital anomalies or aneuploidies, and birthweights were also collected from the medical records. Gravidity, parity, abortion history, and other relevant obstetric details were comprehensively recorded. However, subgroup analysis regarding differences in biomarker levels according to seizure etiologies or semiologies was not conducted due to limited subgroup sizes, restricting meaningful statistical analysis.

### 2.5. Biochemical Analysis

Maternal serum biochemical marker levels were measured from maternal blood samples collected routinely between 15 and 20 weeks of gestation during antenatal visits. Blood samples were collected into serum separator tubes, centrifuged within 30 min at 3000 rpm for 10 min, and stored at −80 °C until analysis. AFP, uE3, and hCG levels were quantitatively measured using a standardized chemiluminescence immunoassay performed with the Roche Cobas^®^ analyzer (Cobas e 411, Roche Diagnostics GmbH, Mannheim, Baden-Württemberg, Germany), following the manufacturer’s protocols. All analyses were conducted in the accredited laboratories, ensuring consistent analytical conditions. Routine quality control measures, including internal quality control samples and external proficiency testing, were performed regularly.

### 2.6. Statistical Analysis

Descriptive statistics are presented as means ± standard deviation for continuous variables and as frequencies and percentages for categorical variables. The normality of the data distribution was assessed using the Kolmogorov–Smirnov and Shapiro–Wilk tests. Comparisons between the epilepsy group and the control group for continuous variables were performed using Student’s *t*-test for normally distributed data and the Mann–Whitney U test for non-normally distributed data. The chi-square test or Fisher’s exact test was used for categorical variables where appropriate. Comparisons among the ASM subgroups (lamotrigine, levetiracetam, and carbamazepine) were performed using one-way ANOVA for normally distributed data and the Kruskal–Wallis test for non-normally distributed data. Propensity score matching was performed based on age, gravidity, parity, abortion history, gestational age at testing, BMI, and smoking status to ensure comparability between the epilepsy and control groups. Matching adequacy was evaluated by comparing standardized differences in the matching variables between the two groups, with differences below 0.1 being considered acceptable. A *p*-value of less than 0.05 was considered statistically significant for all analyses. Statistical analyses were performed using SPSS Statistics for Windows, Version 27.0 (IBM Corp., Armonk, NY, USA).

## 3. Results

### 3.1. Propensity Score Matching Results

Propensity score matching is a successful way to balance groups. The standardized differences for all matching variables were reduced to below 0.1 after matching, indicating that the epilepsy and control groups had similar demographic and clinical characteristics (Table 1).

### 3.2. Demographic and Clinical Characteristics

The final analysis included a total of 86 pregnant women (43 in the ASM-exposed group and 43 in the control group). The demographic and clinical characteristics of the two groups are presented in Table 2. The two groups were similar regarding maternal age, gravidity, parity, abortion history, gestational age at the time of serum screening, BMI, and smoking status (all *p* > 0.05), indicating successful matching based on propensity score matching (all standardized differences < 0.1).

Throughout the pregnancy follow-up, seizure frequency remained stable in most patients. EEG monitoring was performed selectively in six patients (14.0%), revealing no clinically significant EEG changes. Based on the 2025 ILAE classification, focal onset seizures were the most frequent (41.9%), followed by generalized (32.6%) and combined generalized–focal onset seizures (16.3%). Seizure etiology was most commonly genetic (27.9%) or structural (23.3%), while focal onset was the predominant semiology (41.9%). Medication dosage adjustments due to seizure frequency or side effects were necessary in eight patients (18.6%). No serious adverse events requiring hospitalization or emergency department visits related to epilepsy or medication side effects were recorded. During the pregnancy follow-up, none of the patients experienced status epilepticus or were diagnosed with drug-resistant epilepsy. Psychiatric comorbidities were documented in eight patients (18.6%) in the ASM-exposed group, most commonly including anxiety and depressive disorders. Substance abuse disorders were not documented in the medical records. Additionally, there were no hospitalizations or emergency room visits directly related to seizures or antiseizure medication side effects in any of the patients included in the ASM-exposed group.

The normality of maternal serum biomarker distributions was assessed using Kolmogorov–Smirnov and Shapiro–Wilk tests. The Kolmogorov–Smirnov test results indicated non-normal distribution for AFP (*p* = 0.042) and uE3 (*p* = 0.034), while hCG was normally distributed (*p* = 0.127). Similarly, the Shapiro–Wilk tests revealed non-normal distribution for AFP (*p* = 0.038) and uE3 (*p* = 0.029), and normal distribution for hCG (*p* = 0.113).

### 3.3. Antiseizure Medication Types and Daily Doses in Study Group

The types, frequencies, and average daily doses of ASMs used by the participants in the ASM-exposed group are summarized in Table 3. The frequency of ASM use was as follows: levetiracetam (41.9%), lamotrigine (37.2%), carbamazepine (16.3%), and combined lamotrigine + levetiracetam (4.7%).

### 3.4. Comparison of Second-Trimester Maternal Serum Biochemical Markers

The second-trimester maternal serum biochemical marker levels (AFP, uE3, and hCG) in the ASM and control groups are presented in Table 4. Maternal serum AFP (1.34 ± 0.42 vs. 1.01 ± 0.31 MoM; *p* < 0.001) and uE3 (1.28 ± 0.39 vs. 1.05 ± 0.34 MoM; *p* = 0.004) levels were significantly higher in the ASM-exposed group than in the control group. No statistically significant difference was observed between groups in maternal serum hCG levels (1.07 ± 0.46 vs. 1.01 ± 0.42 MoM; *p* = 0.523).

### 3.5. Comparison of Unconjugated Estriol and Alpha-Fetoprotein Values According to Antiseizure Medication Type

The maternal serum biochemical marker levels according to the type of ASM used are summarized in Table 5. No significant differences were observed among the lamotrigine, levetiracetam, and carbamazepine groups regarding maternal serum AFP and uE3 levels (all *p* > 0.05).

### 3.6. Neonatal Outcomes and Birthweights

Detailed anatomical ultrasound and neonatal examinations showed no significant differences in the incidence of fetal congenital anomalies or aneuploidies between the ASM and control groups (ASM-exposed group: one case (2.3%); control group: one case (2.3%); *p* = 1.000). The mean birthweights did not differ significantly between the groups (ASM-exposed group: 3180 ± 410 g; control group: 3250 ± 390 g; *p* = 0.405) (Table 6).

## 4. Discussion

The most important finding of this study is that maternal serum AFP and uE3 levels were significantly increased in pregnant women using ASMs compared with other pregnant women who did not used any medication. However, hCG did not show a significant difference between the groups. Additionally, no evident differences in AFP and uE3 levels were detectable between the different ASM subtypes in the investigation.

Our results are consistent with previous studies showing increased maternal serum AFP and uE3 levels with the use of ASMs in pregnancy [6,7]. In fact, Besimoglu et al. [7] reported significantly increased second-trimester median serum AFP and uE3 levels among pregnant women taking ASMs relative to their cohort controls, which is precisely the same result we found. Similarly, Oksuzoglu et al. [6], based on information from their study, suggested that second-trimester biochemical markers levels were higher among the pregnant women using ASMs in their cohort. This reinforces the findings of our study related to the influence of ASM use on the maternal prenatal screening and outcome data; however, both previous studies included groups of heterogeneous patients, while we used propensity score matching to create well-balanced groups for study comparison to help control for confounding variables and provide more sound findings and conclusions. We summarized the maternal serum biochemical marker values and major findings of prior research together with those from our study in Table 7 to provide a clear overview and facilitate direct comparisons between our findings and previous relevant studies.

**Figure 2:**
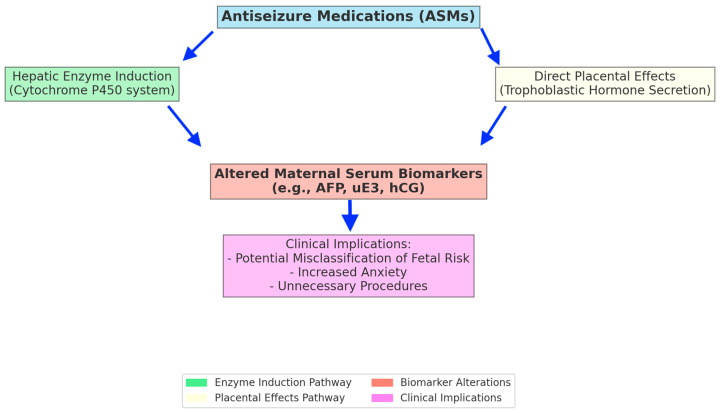
Potential mechanisms by which antiseizure medications affect maternal serum biochemical markers during pregnancy.

The elevated maternal serum AFP and uE3 levels observed in our study may be explained by several pharmacological and physiological mechanisms associated with ASM use. Primarily, certain ASMs, especially carbamazepine, are known to induce hepatic microsomal enzymes, particularly the cytochrome P450 enzyme system, which plays a critical role in hormone metabolism and clearance [11,13]. Increased hepatic enzyme activity could accelerate hormone clearance and trigger compensatory increases in AFP and uE3 synthesis. Although lamotrigine and levetiracetam are generally recognized as weaker enzyme inducers than carbamazepine, these ASMs might still influence hormonal pathways through alternative mechanisms, including the modulation of placental transport functions, indirect effects on maternal liver metabolism, and increased renal clearance during pregnancy due to enhanced renal blood flow and glomerular filtration rate [16,17]. The absence of significant changes in hCG levels in our study aligns with previous findings [6,7]. This could be due to hCG secretion being less susceptible to hepatic enzyme induction and primarily regulated by placental trophoblastic activity, differing mechanistically from steroid hormone synthesis pathways. Similar interpretations were provided by Besimoglu et al. and Oksuzoglu et al., who suggested that different hormonal markers exhibit variable sensitivity to the effects of ASMs, reflecting diverse metabolic and physiological pathways [6,7]. We believe that our findings reflect these multifaceted pharmacological and physiological mechanisms. These potential mechanisms and their clinical implications are clearly illustrated in Figure 2.

In this study, demographic and clinical characteristics were effectively balanced between the ASM and control groups, as demonstrated by standardized differences below 0.1 after propensity score matching. Specifically, critical confounding factors such as maternal age, gravidity, parity, abortion history, gestational age at testing, BMI, and smoking status were carefully matched to ensure comparability between the groups. Previous studies indicate that these demographic and clinical variables can significantly affect maternal serum biochemical marker levels during pregnancy [18,19,20]. Therefore, achieving balanced groups was essential to confidently attributing the observed differences in biochemical markers primarily to ASM usage rather than confounding variables.

In addition to biochemical alterations, our study carefully assessed clinical epilepsy management and related outcomes throughout pregnancy. Our findings demonstrate that seizure frequency remained stable for most patients, supporting the effectiveness of consistent ASM management in controlling epilepsy during pregnancy. The selective EEG monitoring performed in our study revealed no clinically significant changes, which is consistent with previous studies [3,4] indicating that EEG abnormalities typically remain stable with effective treatment. The medication dosage adjustments required in 18.6% of patients due to seizure control or side effects align with earlier reports emphasizing individualized pharmacological management to maintain optimal seizure control during pregnancy [2,17]. Importantly, we did not observe any instances of status epilepticus or drug-resistant epilepsy, nor did we document significant psychiatric comorbidities or substance abuse disorders among our participants, indicating careful patient selection and close clinical monitoring. Furthermore, the absence of hospitalizations or emergency room visits related to seizures or medication side effects further underscores the importance and effectiveness of regular follow-ups and tailored epilepsy management during pregnancy [21].

In addition to the biochemical alterations, our study comprehensively evaluated clinical epilepsy management and seizure characteristics during pregnancy. Seizure frequency remained stable in most patients, supporting the effectiveness of consistent ASM therapy. According to the 2025 ILAE classification, focal onset seizures were the most common, followed by generalized and combined generalized–focal types, which aligns with seizure distributions reported in reproductive-age women [1,3,15]. EEG monitoring was selectively performed in clinically indicated cases and revealed no significant abnormalities, consistent with prior evidence suggesting EEG patterns remain stable under effective treatment [3,4]. Psychiatric comorbidities, primarily anxiety and depression, were observed in 18.6% of patients, which is consistent with the known neuropsychiatric burden in women with epilepsy [1]. No cases of drug-resistant epilepsy, status epilepticus, or substance use disorders were identified, and no epilepsy-related hospitalizations or emergency department visits occurred. These findings underscore the importance of individualized management and close monitoring during pregnancy to ensure favorable maternal neurological and psychiatric outcomes [2,17,21].

In the present study, levetiracetam (41.9%) and lamotrigine (37.2%) were the most commonly prescribed ASMs, followed by carbamazepine (16.3%), while a small proportion (4.7%) used combined therapy (lamotrigine and levetiracetam). These findings align with current clinical guidelines that recommend levetiracetam and lamotrigine as first-line ASM treatments during pregnancy due to their favorable safety profiles compared to older ASMs such as valproic acid or carbamazepine [21,22,23]. None of the participants used valproic acid, consistent with contemporary recommendations discouraging its use during pregnancy. Only ASM therapies used consistently for at least three months prior to the screening test were included, minimizing potential confounding due to recent medication changes. The average daily doses of medications remained within therapeutic ranges reported in the literature [24].

Our subgroup analysis revealed no significant differences in maternal serum AFP and uE3 levels among the different ASM types (lamotrigine, levetiracetam, and carbamazepine), consistent with previous reports [6,7]. Although carbamazepine is known as a stronger hepatic enzyme inducer, the limited subgroup sample sizes may have restricted the ability to detect such differences. These findings highlight the need for cautious interpretation of second-trimester maternal serum screening results in ASM-exposed pregnancies, as elevated maternal serum AFP and uE3 levels may lead to potential misclassification of fetal risk and unnecessary invasive procedures. Incorporating maternal medication history into risk assessment algorithms may improve the accuracy of prenatal screening and optimize counseling strategies for pregnant women with epilepsy.

One of the primary strengths of our study is the use of rigorous propensity score matching, which significantly enhanced the robustness, internal validity, and reliability of our observed associations by ensuring balanced demographic and clinical characteristics between groups. Moreover, our study included a clearly defined ASM-exposed population with consistent medication use for at least three months prior to the second-trimester screening test, minimizing potential confounding effects from medication changes during pregnancy. Additionally, the multicentric design involving both tertiary and secondary care centers increases the generalizability of our findings. However, caution should still be exercised when generalizing results beyond populations similar to those included in this study, particularly given the specific patient characteristics and clinical management practices in our study centers. Lastly, our study provides detailed information on the types and dosages of the ASMs used, reflecting current clinical practices and enhancing the relevance of our results for clinical settings.

Our study has several limitations that need to be considered when interpreting the findings. First, the retrospective nature of our research inherently limits the control over data quality and completeness. Second, despite careful matching, the relatively small sample size may have restricted the statistical power to detect subtle but clinically meaningful differences, especially within individual ASM subgroups. Furthermore, the limited number of patients within each ASM type subgroup prevented meaningful analyses of potential dose-dependent effects of ASMs on maternal serum biochemical markers. Additionally, the combined-therapy subgroup (lamotrigine + levetiracetam) could not be included in the subgroup analyses due to an insufficient number of patients. Due to the retrospective design of our study, detailed information regarding concomitant medications and other potential clinical conditions that might influence maternal serum biomarker levels was limited. Although we applied rigorous propensity score matching to control for major demographic and clinical variables (maternal age, BMI, smoking status, parity, gravidity, abortion history, and gestational age), residual confounding from other unrecorded factors such as dietary supplements, additional medications, or subtle medical conditions cannot be completely ruled out. Although neonatal outcomes including congenital anomalies and birthweights were reported, the retrospective design limits detailed assessments of subtle clinical factors and long-term developmental outcomes. Lastly, the study lacked long-term neonatal outcome data, limiting our ability to correlate biochemical marker changes directly with clinical pregnancy outcomes or fetal complications.

Future studies should aim to address the limitations identified in our research by utilizing larger, prospective cohorts to validate these findings and enhance statistical power, especially for subgroup analyses. Additionally, research designed to specifically investigate the dose-dependent effects of individual ASMs on maternal biochemical marker levels could significantly contribute to our understanding of medication-specific risks during pregnancy. Moreover, longitudinal studies assessing not only biochemical markers but also detailed neonatal and long-term neurodevelopmental outcomes would provide more comprehensive insights into the clinical relevance of these biochemical alterations. Ultimately, such research could help refine prenatal screening guidelines and optimize management strategies for pregnant women with epilepsy.

## 5. Conclusions

In conclusion, the maternal use of ASMs during pregnancy significantly elevates second-trimester maternal serum AFP and uE3 levels, potentially impacting the interpretation of prenatal screening tests. Clinicians should carefully consider medication exposure when evaluating these biochemical markers in pregnant women with epilepsy to prevent the misclassification of fetal risks and avoid unnecessary invasive procedures. Future larger-scale, prospective studies are needed to confirm these findings and further refine prenatal management strategies in ASM-exposed pregnancies.

## Figures and Tables

**Table 1:** Standardized differences for variables before and after propensity score matching.

Variables	Standardized Difference (Before Matching)	Standardized Difference (After Matching)
Maternal age	0.25	0.03
Gravidity	0.21	0.02
Parity	0.19	0.04
Abortion history	0.15	0.01
Gestational age at testing	0.22	0.03
BMI	0.31	0.05
Smoking status	0.17	0.00

BMI: body mass index. Standardized difference values less than 0.1 indicate well-balanced variables between groups after matching.

**Table 2:** Demographic and clinical characteristics of antiseizure medication and control groups.

Variables	ASM-Exposed Group (*n* = 43)	Control Group (*n* = 43)	*p*-Value
Maternal age (years)	29.6 ± 5.2	29.2 ± 4.9	0.712
Gravidity	2.4 ± 1.3	2.3 ± 1.4	0.825
Parity	1.2 ± 0.8	1.1 ± 0.7	0.615
Abortion history	0.3 ± 0.6	0.3 ± 0.5	0.893
Gestational age at testing (weeks)	17.6 ± 1.2	17.8 ± 1.1	0.423
BMI (kg/m^2^)	26.1 ± 4.0	25.7 ± 3.8	0.648
Smoking status, *n* (%)			1.000
Smoker	6 (14.0%)	6 (14.0%)	
Non-smoker	37 (86.0%)	37 (86.0%)	

ASM: antiseizure medication; BMI: body mass index. Data presented as numbers (%) or means ± standard deviation (SD).

**Table 3:** Types, frequencies, and daily doses of antiseizure medications.

Antiseizure Medication	Patients, *n* (%)	Daily Dose, Mean ± SD (mg/day)	Dose Range (mg/day)
Levetiracetam	18 (41.9%)	1380 ± 510	500–3000
Lamotrigine	16 (37.2%)	210 ± 85	50–400
Carbamazepine	7 (16.3%)	640 ± 210	400–1200
Lamotrigine + levetiracetam (combined)	2 (4.7%)	Lamotrigine: 175 ± 35	150–200
		Levetiracetam: 1250 ± 350	900–1600

Data presented as numbers (%) or means ± standard deviation (SD).

**Table 4:** Second-trimester maternal serum biochemical marker levels.

Marker (MoM)	ASM-Exposed Group (*n* = 43)	Control Group (*n* = 43)	Mean Difference (95% CI)	*p*-Value
AFP	1.34 ± 0.42	1.01 ± 0.31	0.33 (0.17–0.49)	<0.001
uE3	1.28 ± 0.39	1.05 ± 0.34	0.23 (0.08–0.38)	0.004
hCG	1.07 ± 0.46	1.01 ± 0.42	0.06 (−0.14–0.26)	0.523

ASM: antiseizure medication; AFP: alpha-fetoprotein; hCG: human chorionic gonadotropin; MoM: multiples of median; uE3: unconjugated estriol; CI: confidence interval. Data presented as means ± standard deviation (SD).

**Table 5:** Maternal serum unconjugated estriol and alpha-fetoprotein levels according to antiseizure medication type.

Marker (MoM)	Lamotrigine (*n* = 16)	Levetiracetam (*n* = 18)	Carbamazepine (*n* = 7)	f-Value	*p*-Value
AFP	1.29 ± 0.34	1.32 ± 0.38	1.39 ± 0.37	0.98	0.384
uE3	1.23 ± 0.33	1.27 ± 0.35	1.31 ± 0.36	0.24	0.785

AFP: alpha-fetoprotein; uE3: unconjugated estriol; MoM: multiples of median. Data presented as mean ± standard deviation (SD).

**Table 6:** Neonatal outcomes and birthweights of the study groups.

Variable	ASM-Exposed Group (*n* = 43)	Control Group (*n* = 43)	*p*-Value
Congenital anomaly/aneuploidy, *n* (%)	1 (2.3%)	1 (2.3%)	1.000
Birthweight (grams, mean ± SD)	3180 ± 410	3250 ± 390	0.405

ASM: antiseizure medication. Data presented as means ± standard deviation (SD).

**Table 7:** Comparison of maternal serum biochemical markers in the present and previous studies investigating the effects of antiseizure medications on second-trimester screening test parameters.

Study (Year)	Study Type	Medication Evaluated	Participants (ASM-Exposed Patients vs. Controls)	AFP (MoM)	uE3 (MoM)	hCG (MoM)	Major Findings
Present study (2025)	Retrospective cohort	Lamotrigine, Levetiracetam, and Carbamazepine	43 vs. 43	1.34 ± 0.42	1.28 ± 0.39	1.07 ± 0.46 (NS)	Elevated AFP and uE3; no change in hCG
Öksüzoğlu et al. (2020) [5]	Retrospective case–control	Various ASMs (including carbamazepine and valproate)	122 vs. 122	0.99 ± 0.5	1.5 ± 0.7	1.24 ± 0.6 (NS)	Elevated AFP and uE3; no change in hCG
Besimoğlu et al. (2022) [6]	Retrospective cohort	Multiple ASMs	53 vs. 106	1.28 ± 0.47	1.24 ± 0.42	0.97 ± 0.38 (NS)	Elevated AFP and uE3; no change in hCG

AFP: alpha-fetoprotein; uE3: unconjugated estriol; hCG: human chorionic gonadotropin; ASM: antiseizure medication; MoM: multiples of median; NS: non-significant.

## Data Availability

The data supporting the findings of this study are available from the corresponding author upon reasonable request.

## Abbreviations

The following abbreviations are used in this manuscript:

ASM	Antiseizure medication
AFP	Alpha-fetoprotein
ALT	Alanine aminotransferase
AST	Aspartate aminotransferase
BMI	Body mass index
hCG	Human chorionic gonadotropin
CPR	Cerebroplacental Ratio
MoM	Multiples of median
AC	Abdominal Circumference
uE3	Unconjugated estriol
EEG	Electroencephalography
ILAE	International League Against Epilepsy
